# A Journey to the Conformational Analysis of T-Cell Epitope Peptides Involved in Multiple Sclerosis

**DOI:** 10.3390/brainsci10060356

**Published:** 2020-06-08

**Authors:** Catherine Koukoulitsa, Eleni Chontzopoulou, Sofia Kiriakidi, Andreas G. Tzakos, Thomas Mavromoustakos

**Affiliations:** 1 Department of Chemistry, National and Kapodistrian University of Athens, Zographou, 15784 Athens, Greece; ckoukoulitsa@chem.uoa.gr (C.K.); elenichontzo@chem.uoa.gr (E.C.); sofki@chem.uoa.gr (S.K.); 2 Department of Chemistry, Section of Organic Chemistry and Biochemistry, University of Ioannina, 45110e Ioannina, Greece; atzakos@uoi.gr

**Keywords:** conformational analysis, peptides, altered peptide ligands, multiple sclerosis, MS, NMR spectroscopy, NOE-constraints, molecular dynamic, trimolecular complex, experimental autoimmune encephalomyelitis

## Abstract

Multiple sclerosis (MS) is a serious central nervous system (CNS) disease responsible for disability problems and deterioration of the quality of life. Several approaches have been applied to medications entering the market to treat this disease. However, no effective therapy currently exists, and the available drugs simply ameliorate the destructive disability effects of the disease. In this review article, we report on the efforts that have been conducted towards establishing the conformational properties of wild-type myelin basic protein (MBP), myelin proteolipid protein (PLP), myelin oligodendrocyte glycoprotein (MOG) epitopes or altered peptide ligands (ALPs). These efforts have led to the aim of discovering some non-peptide mimetics possessing considerable activity against the disease. These efforts have contributed also to unveiling the molecular basis of the molecular interactions implicated in the trimolecular complex, T-cell receptor (TCR)–peptide–major histocompatibility complex (MHC) or human leucocyte antigen (HLA).

## 1. Introduction

Multiple sclerosis (MS) is a serious disease of the central nervous system (CNS). MS affects almost 3.3 million people worldwide [1]. It affects more females than males between the ages of 20 and 40 [2]. MS-related disability significantly affects the quality of life (e.g., restraints on daily life activities) [3]. As the number of patients continuously increases, negative effects on social and economic aspects have been observed [4,5]. Factors such as genetic, environment, metabolism and viral infections considerably progress the disease [6,7].

MS is classified into four subclasses according to the increase of the neurologic deterioration of the disease:

1. Relapsing-remitting MS (RRMS): This is the most frequently occurring and affects ca. 85% of all MS patients. The patients with RRMS suffer from relapses and remissions of their neurological symptoms.
2. Secondary progressive MS (SPMS): This follows the development of RRMS and causes further worsening of the disease.
3. Primary progressive MS (PPMS): This affects 8–10% of patients and is characterized by the gradual further worsening of the disease.
4. Progressive-relapsing MS (PRMS): This is the least often occurring class, affecting less than 5% of patients and progressing from onset [8,9,10].

**Figure 1:**
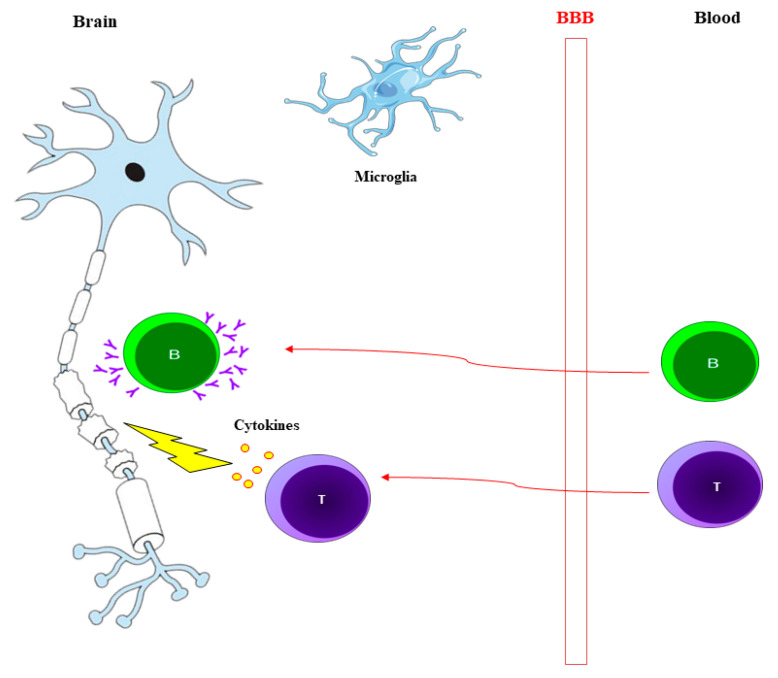
T-cells enter the blood brain barrier (BBB) and release cytokines which degrade the myelin. The cytokines can also recruit some other cells as B-cells. These cells enter the BBB and produce antibodies which target the myelin for further degradation. Activated microglia are also involved in myelin degradation.

MS takes place in brain and spinal cord regions containing myelin. As shown in Figure 1, MS lesions involve demyelination and inflammation of B-cells, T-cells, macrophages and activated microglia. Then follows tissue damage, which includes loss of neurons and oligodendrocytes, astrogliosis and remyelination [11,12].

The cause of autoimmune disease MS is still mostly unknown. It is hypothesized that environment induces MS in individuals prone to the disease. The molecular mimicry theory has been used to explain the pathogenesis of MS. The gathered evidence proposes that viral peptidic epitopes bearing sequence homology to protein regions of normal human tissue are responsible for the initiation of the disease. The immune response of T-cells targets mainly the viral epitopes. However, cross-reaction with the normal human tissue leads to the autoimmune disease [13,14].

The myelin basic protein (MBP), the proteolipid protein (PLP), the myelinoligodendrocyte glycoprotein (MOG), and the myelin associated oligodendrocytic basic protein (MOBP), have been associated as T-cell epitopes in MS. These peptides have been utilized to trigger experimental autoimmune encephalomyelitis (EAE). EAE is the most frequently and broadly used animal model that simulates MS [15,16,17,18,19,20,21].

Although advances in MS treatment have proceeded impressively, the currently available medications are not fully in line to respond to the future and emerging needs raised by the complicated nature of MS [22].

One of the major approaches for the treatment of MS is the peptidic or peptidomimetic therapeutic approach [23,24]. There are different steps involved in the development of peptidomimetic drugs in a rational design strategy. In the first step the minimal peptide amino acid sequence that exerts the activity (epitope) and serves as a lead compound is identified. In the second step the information derived from nuclear magnetic resonance (NMR) spectroscopy, and/or molecular modeling and/or x-ray crystallography is utilized in order to define a putative bioactive conformation of the minimal peptide sequence [25]. In the third step the resultant 3D architecture is used for the development of non-peptide mimetics that are prone to metabolic clearance.

Activated encephalitogenic T-cells, triggered by the formation of a trimolecular complex between the T-cell receptor (TCR), the peptide (antigen)—with identical residue sequence to a fragment of a protein of the myelin sheath—and the major histocompatibility complex (MHC) or human leukocyte antigen (HLA), initiate the onset of MS. The potential of the peptide–HLA complex to activate T-cells parallels the strength of its binding affinity with TCR [26,27,28]. It follows the stimulation, or not, of T-cells that cause MS [29,30,31,32,33].

The dimer HLA class II receptors contain two polypeptide chains named as α and β [34,35]. Their joined polypeptide chains form a single receptor suitable to form a complex with the antigen binders. This complex is recognized by the T-cell receptors on the cell surface. The formed trimolecular complex leads to the activation of T-cells through a series of biochemical alterations and the triggering of the immune response to the antigen [36].

This review summarizes the conformational analysis of peptides involved in multiple sclerosis. In addition the impact of these conformational changes on rational drug design is described.

## 2. Results and Discussion

Mouzaki et al. [37] pointed out that peptides constitute a class of administered molecules as immunomodulatory drugs due to their rapid and cost-effective synthesis. The peptides that can cause EAE in animals are called agonists and those that can compete the action of the agonists and treat EAE are called antagonists.

In the discussed studies peptides are used that either map to wild-type MBP, PLP [38] or MOG epitopes or are mutants (altered peptide ligands, APLs), which are linear or cyclized variants that are more resistant to in vivo enzymatic degradation [39]. APLs differ from their parent encephalitogenic peptides by single amino acid substitutions and can inhibit autoimmune mediated disease through several mechanisms.

For many years we have made an effort to explore the conformational properties that govern various epitopes related to EAE with their agonist and antagonists both in solution and in trimolecular complexes (drug:TCR:HLA). In this review we will outline the most significant results obtained from these studies.

**Figure 2:**
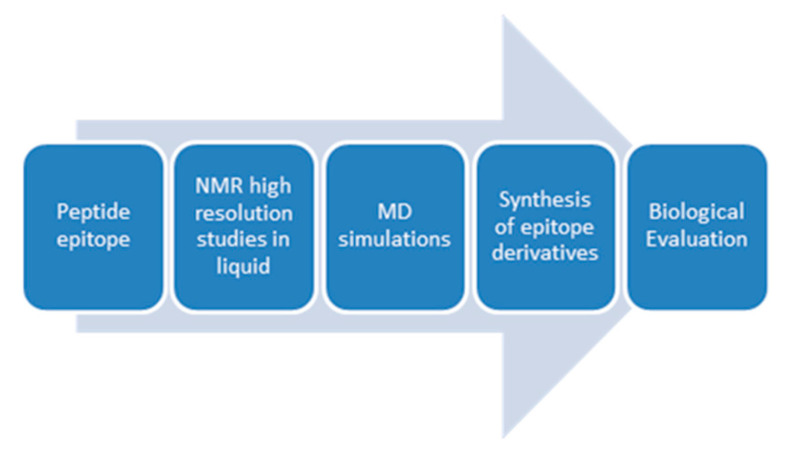
Steps of a rational design process aiming to aid the development of peptide mimics against multiple sclerosis (MS).

The first step in these studies is to extract favored averaged conformations of the epitopes in solution using NMR spectroscopy. These conformations after energy minimization serve as initial conformations for applying molecular dynamics (MD) simulations in the generation of the trimolecular complex. The results will lead to the synthesis of antagonist peptides which could potentially provide useful mechanistic information to combat MS (Figure 2).

The conformational analysis of hMOG_35–55_ epitope (Met_35_-Glu-Val-Gly-Trp-Tyr-Arg-Pro^42^-Pro-Phe-Ser-Arg-Val-Val-His-Leu-Tyr-Arg-Asn-Gly-Lys_55_) and its mutants (hMOG_35–55_(Ala^41^) and hMOG_35–55_(Ala^41,46^)) alone and in the trimolecular complex containing HLA and TCR have been studied using MD simulations [36]. The results showed that the hMOG_35–55_ epitope in the MD trajectory does not retain the linear conformation. Its dominant conformation shows two bends in the polypeptide backbone between residues Trp^39^, Tyr^40^ and Arg^41^ and Val^48^ and Arg^52^.

**Figure 3:**
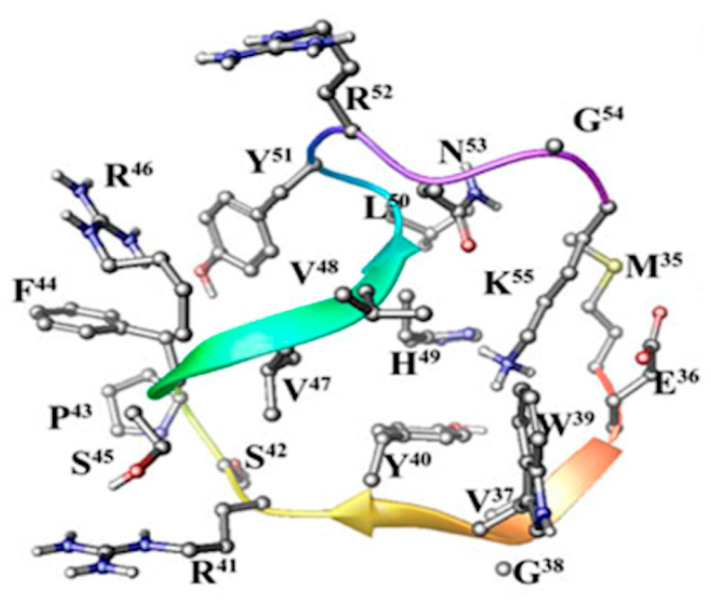
Low energy conformer of hMOG_35-55_ (myelinoligodendrocyte glycoprotein) derived from in silico molecular synamics (MD) calculations restricted with nuclear Overhauser effect (NOE)-constraints.

This conformation is similar to that published for the rat/mouse MOG_35–55_ peptide by Ntountaniotis et al. [40] in DMSO and D_2_O solvents (Figure 3).

**Figure 4:**
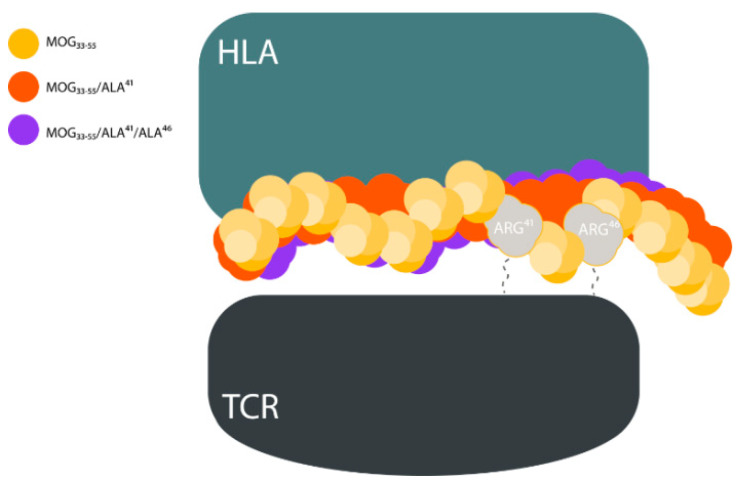
The replacement of Arg^41^ or Arg^41^ and Arg^46^ of hMOG_35–55_ with Ala interrupts the hydrogen bonding (HB) with the amino acids Asp^98^, Ser^101^, and Asn^104^ of T-cell receptors (TCR). This may be due to the decrease of polarity of Ala vs. Arg (disruption of the interaction network) and may lead to a reduced bending of Ala in the low energy conformation of hMOG_35–55_. HLA—Human Leukocyte Antigen.

During the formation of the trimolecular complex the amino acids Arg^41^ and Arg^46^ of hMOG_35–55_ anchor at TCR and Tyr^40^ interacts with HLA. The amino acids Arg^41^ and Arg^46^ form an extensive hydrogen bonding (HB) network with both receptors. Substitution of Arg^41^ or Arg^41^ and Arg^46^ with Ala leads to the two mutants hMOG_35–55_ (Ala^41^) and hMOG_35–55_ (Ala^41^, Ala^46^). These mutations lead to the elimination of key interactions with TCR but leave intact the binding affinity towards the HLA receptor. These two mutants function as EAE inhibitors. This finding is significant as it provides basic mechanistic aspects of the action of agonist versus antagonist peptides (Figure 4).

The conformational analysis of MBP_77–89_ and the antagonist altered ligands (Arg^91^, Ala^96^) MBP_87–99_ and (Ala^91,96^) MBP_87–99_ have been studied. All the three molecules showed an extended conformation in DMSO environment with no long-range nuclear Overhauser effects (NOEs) [41] in disagreement with the observations recorded in other chemical environments [29].

Interestingly, X-ray results existed for a peptide analogue of MBP_87–99_ that formed a trimolecular complex with a human TCR and HLA-DR2b [42]. A bioactive conformation of APL that resembled that of the crystallized peptide was derived from the molecular dynamics trajectories (Root-Mean Square Deviation (RMSD) value of 0.95 Å). The two peptides were oriented similarly to the two TCR anchor residues, His^88^ and Phe^89^, and the HLA anchor residue Phe^90^.

**Figure 5:**
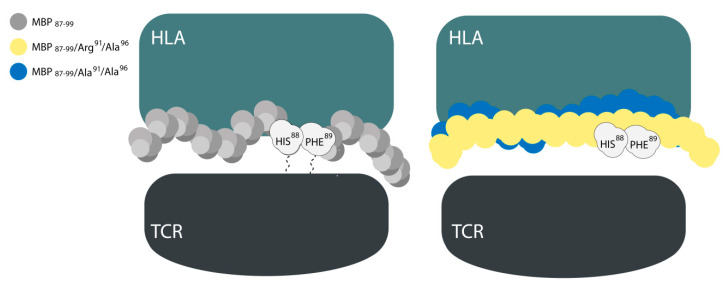
(**left**) His^88^ and Phe^89^ of hMBP_87–99_ (myelin basic protein) interact with the TCR receptor. (**right**) In the two antagonists (Arg^91^, Ala^96^) MBP_87–99_ and (Ala^91,96^) MBP_87–99_ this interaction is lost as the two amino acids are buried in HLA grooves.

These two amino acids orient variably in the trimolecular complex for (Arg^91^, Ala^96^) MBP_87–99_ and (Ala^91,96^) MBP_87–99,_ and remain buried in HLA grooves and cannot interact with the TCR. This finding may explain the antagonism of the two altered ligands (Figure 5).

**Figure 6:**
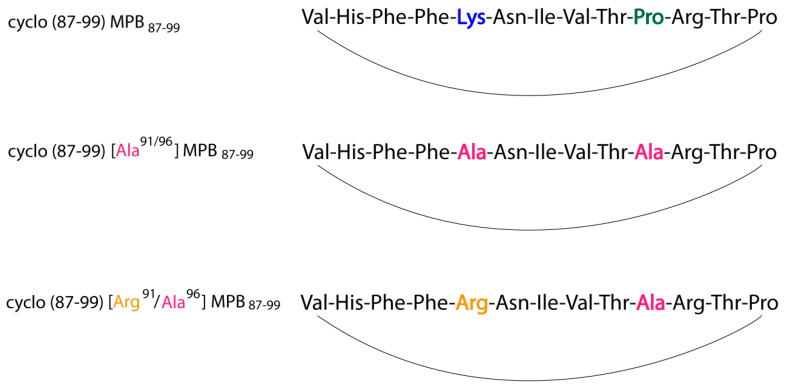
Structures of cyclo(91–99)(Ala^96^)MBP_87–99_, cyclo(87–99)(Ala^91^,^96^)MBP_87–99_ and cyclo(87–99)(Arg^91^, Ala^96^)MBP_87–99_.

The cyclo (91–99)(Ala^96^)MBP_87–99_, cyclo(87–99)(Ala^91,96^)MBP_87–99_ and cyclo(87–99)(Arg^91^, Ala^96^)MBP_87–99_ (Figure 6), except the wild-type linear MBP_87–99_, were found to strongly inhibit MBP_72–85_- induced EAE in Lewis rats. Cyclo(87–99)(Arg^91^, Ala^96^)MBP_87–99_ provided long protection for the EAE induction [39,43,44].

**Figure 7:**
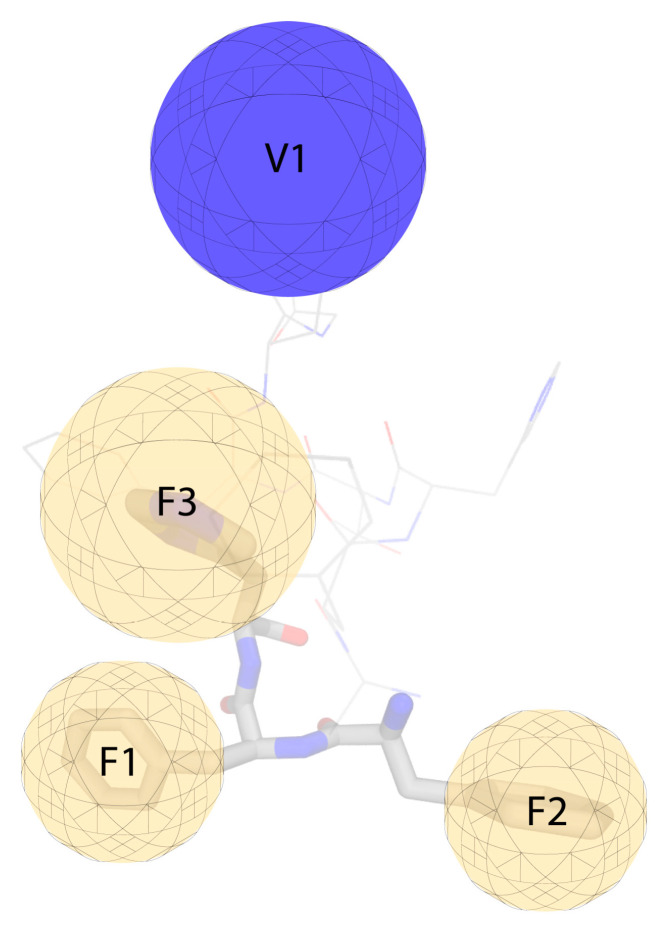
Pharmacophore model depicted using the conformational properties obtained from the conformational analysis for the cyclic altered peptide ligands (APLs). Exclusion volume V1 is presented with a blue sphere, feature F1 (Phe^90^), F2 (Phe^89^), F3 (Phe^88^) with a yellow sphere.

Conformational analysis was achieved for the three cyclo(87–99) MBP_87–99_, cyclo(87–99) (Ala^91,96^) MBP_87–99_, and cyclo(87–99) (Arg^91^, Ala^96^) MBP_87–99_ analogs using 2D NMR spectroscopy and computational analysis. The conformational analysis of the three synthetic analogues showed that their bioactivity, or its absence, may be attributed to the distinct local conformation, overall topology and exposed area after binding with MHC II. An overall larger solvent accessible area may occlude the approach and binding of the TCR on the APL-MHC complex. In contrast, more compact structures do not block weak interactions as TCR approaches and can induce EAE antagonism. These results led to the generation of the pharmacophore model described in Figure 7 [45].

Two citrullinated peptides, the linear (Cit^91^, Ala^96^, Cit^97^)MBP_87–99_ and cyclo(87–99)(Cit^91^, Ala^96^, Cit^97^)MBP_87–99_ have been synthesized by citrullinating the Arg residues 91 and 97 in the antagonists, linear (Arg91, Ala96)MBP_87–99_ and cyclo(87–99)(Arg^91^, Ala^96^)MBP_87–99_ peptides. In contrast to the antagonists, these citrullinated molecules induced EAE. Molecular modeling results pointed out that both Cit^91^ and Cit^97^ residues are oriented toward the TCR and possibly are interacting with the complementarity-determining region (CDR3) loops of the TCR, thus triggering an altered cytokine response [46].

Another epitope which is shown to induce EAE in guinea pigs is the linear peptide MBP_74–85_ (Gln^1^-Lys^2^-Ser^3^-Gln^4^-Arg^5^-Ser^6^-Gln^7^-Asp^8^-Glu^9^-Asn^10^-Pro^11^-Val^12^-NH_2_). A Rotating frame Overhauser Effect Spectroscopy (ROESY) connectivity was observed for the molecule in DMSO between αVal^12^-αGln^1^, suggesting a cyclic conformation. This intriguing result prompted the synthesis of the cyclic analogue by tethering the εNH_2_ of Lys and γCOOH of Glu at positions 2 and 9, respectively. Cyclic peptides are well known to be more stable and less susceptible to enzymatic degradation than linear peptides. Moreover, cyclic peptides are an important intermediate step in the rational design and development of non-peptide mimetics [47].

**Figure 8:**
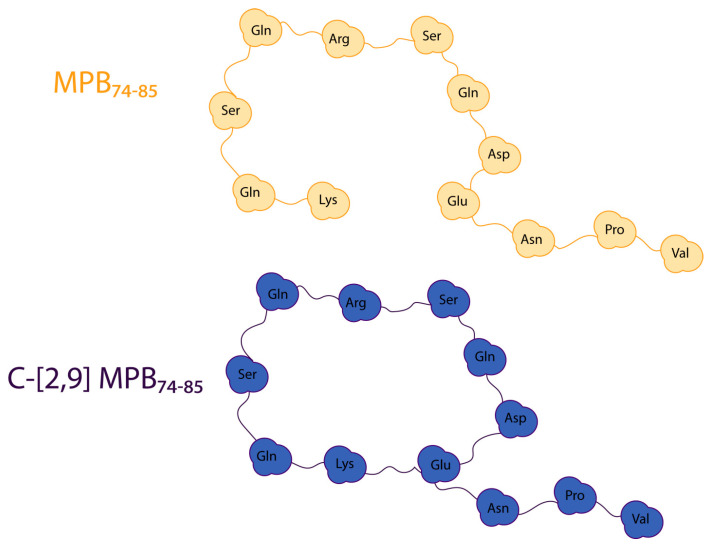
2D and 3D models of linear MBP_74-85_ (**top**) and its cyclic analogue (**bottom**).

This cyclic analogue illustrated comparable bioactivity with the linear one, confirming that the possible bioactive conformation of MBP_74–85_ resembles that of the cyclic variant or the cyclic variant resembles more, from the available ensemble, the structure of the linear peptide that is of biological significance. The structures of the linear and cyclic analogues are shown in Figure 8. The same relationship was observed with the linear Ala^81^ MBP_74–85_ and its cyclic analogue [25].

Tzakos et al. [48] applied NMR and molecular dynamic simulations to study the conformational properties of agonist MBP, Gln^74^-Lys^75^-Ser^76^-Gln^77^-Arg^78^-Ser^79^-Gln^80^-Asp^81^-Glu^82^-Asn^83^-Pro^84^-Val^85^ (MBP_(74–85)_), and its antagonist analogue Ala^81^MBP_(74–85)_. The agonist MBP_(74–85)_ adopted a compact conformation attributed to electrostatic interactions of Arg^78^ with the side chains of Asp^81^ and Glu^82^. Arg^78^ adopted a well-defined conformation, which did not depend on the solvent. Such electrostatic interactions were not observed in the antagonist Ala^81^ MBP_(74–85)_, and a high flexibility of the side chain of Arg^78^ was observed. The positively charged residue Arg^78^ is suggested to stabilize the local microdomains (epitopes) of the integral protein. Flexible docking calculations point out that Gln^74^, Ser^76^ and Ser^79^ are MHC II anchor residues. Lys^75^, Arg^78^ and Asp^81^ are the mainly solvent-exposed residues and this may signify their participation in the formation of the trimolecular T-cell receptor–MBP_(74–85)_–MHC II complex.

In another study the conformational analysis of the immunodominant epitope of acetylated myelin basic protein residues 1–11 (Ac-MBP1–11) and its ALPs, mutated at position 4 to an alanine (Ac-MBP1–11(4A)) or a tyrosine residue (Ac-MBP1–11(4Y)), was achieved. The amino acids constituting the MBP^1–11^ are Ala-Ser-Gln-Lys-Arg-Pro-Ser-Gln-Arg-His-Gly (Ac-MBP1–11). The Ac-MBP1–11(4A) analogue inhibited EAE symptoms induced by encephalitogenic Ac-MBP1–11 epitope when co-injected in (PL/J × SJL)F1 mice. These results are interpreted to suggest that Ac-MBP1–11(4A) induced immunomodulation that inhibits EAE in vivo [49]. Studies indicated that the residue at position 4 in MBP1–11 peptide plays a major role in binding of the peptide to MHC class II, I–Au [50,51]. The mutated analogue Ac-MBP1–11(4A) binds to I–Au with a minimum of 50-fold higher affinity in comparison to the native Ac-MBP1–11 [52]. In addition, the mutation at position 4 of Lys to Tyr (Ac-MBP1–11(4Y)) increases the stability of the I–Au-peptide complex by enhancing 1500-fold the affinity, which triggers Ac-MBP1–11 T-cells more effectively in relation to Ac-MBP1–11(4A) [53].

The conformational analysis of the three analogues showed that they adopt an extended conformation in deuterated DMSO solvent due to the absence of long-distance NOEs. Furthermore, they adopt a similar conformation when bound to the active site of the MHC II. Gln^3^ residue is a TCR contact site and has a different orientation in the mutated analogues. Specifically, its side chain is not solvent exposed, and it is not available for interaction with the TCR. The main MHC contact residues (Ser^2^, Pro^6^ and Ser^7^) stand in the same position for all peptides [54].

The conformational properties of MBP_83–99_ have been studied using NMR spectroscopy in DMSO to simulate the biological environment. The results showed that the peptide exists in a rather extended conformation and forms a helix between Val^87^ and Phe^90^ [55].

**Figure 9:**
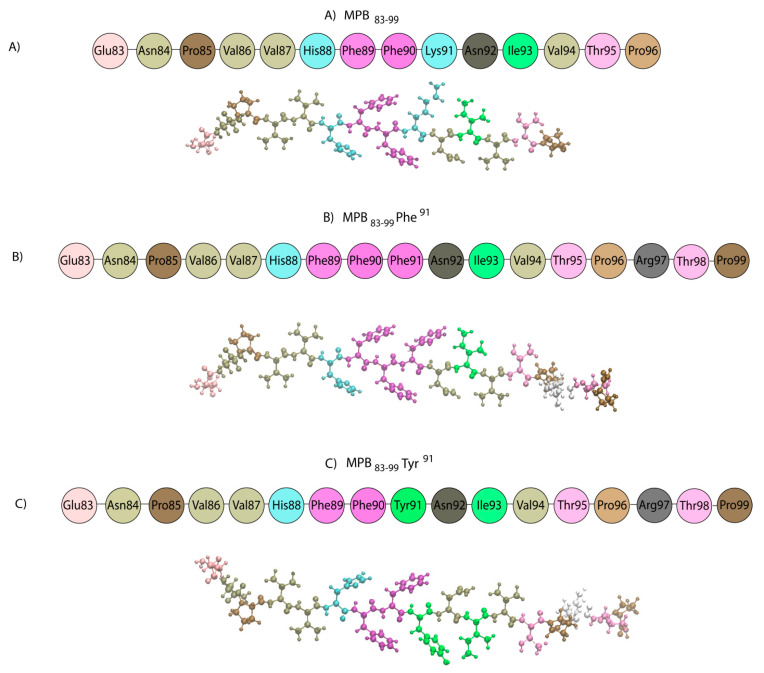
Sequences and 3D low energy structures of (**A**) MBP_83–96_ (numbering is according to the human MBP_83–99_ epitope) and the two synthetic analogs (**B**) MBP_83–99_ (Phe^91^) and (**C**) MBP_83–99_ (Tyr^91^).

Two analogues of the MBP_83–99_ epitope substituted at Lys^91^ (primary TCR contact) with Phe (MBP_83–99_ (Phe^91^)) or Tyr (MBP_83–99_ (Tyr^91^)) were synthesized (Figure 9).

The two analogues showed distinct antagonistic activity versus the agonistic activity of the MBP_83–99_ epitope. The conformational analysis of the two APLs was performed using NMR spectroscopy and MD. Both synthetic analogues show an extended conformation in agreement with the structural features of the peptides that interact with the HLA-DR2 and TCR receptors. MD simulations of the two analogues in complex with HLA-DR2 (DRA, DRB1*1501) and TCR revealed their modes of interactions. MBP_83–99_ (Phe^91^) analogue adopts more interactions during the formation of the trimolecular complex relatively to MBP_83–99_ (Tyr^91^), as their trajectory profiles confirmed. This may explain the improved biological profile of the latter. The two analogues differ in the way of binding relatively to the wild epitope MBP_83–96_. This is attributed to the fact that mutation of Lys^91^ by either Tyr or Phe alters their stereoelectronic properties.

This alteration of the stereoelectronic properties affects the binding mode of the regional amino acids and explains their antagonistic or agonistic activity. Such binding mode differences have been observed and outlined above with the MBP_87–99_ epitope [45,56,57,58,59,60].

**Figure 10:**
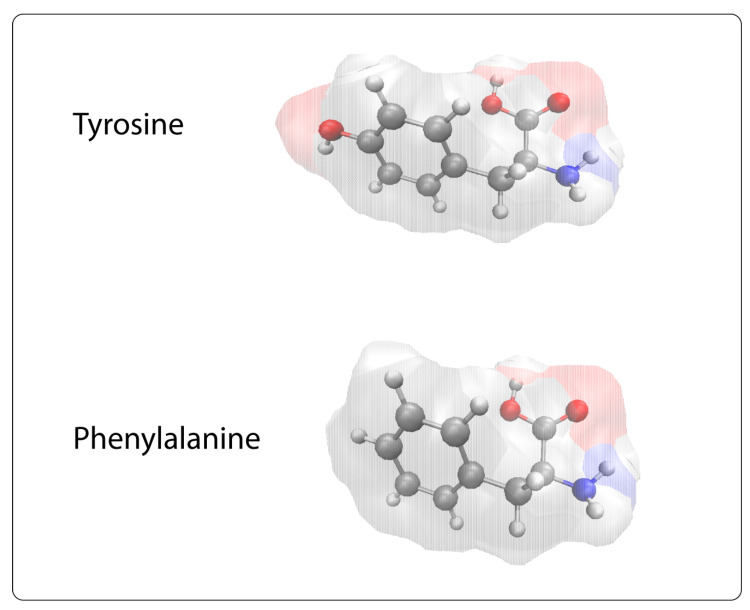
The absence of the phenolic hydroxyl group in Phe is responsible for the different biological properties between the two synthetic analogues MBP_83–99_ (Tyr^91^) and MBP_83–99_ (Phe^91^).

It is important to note that although the two peptides mentioned above differ only in a small segment, they possess distinct biological profiles. The tyrosine^91^ in MBP_83–99_ (Tyr^91^) possesses a phenolic hydroxyl group that induces differential biological activity. This is in agreement with a plethora of literature data pointing out the key role of the phenolic group in drug bioactivity [61,62,63,64,65,66,67,68,69,70,71,72] (Figure 10).

**Figure 11:**
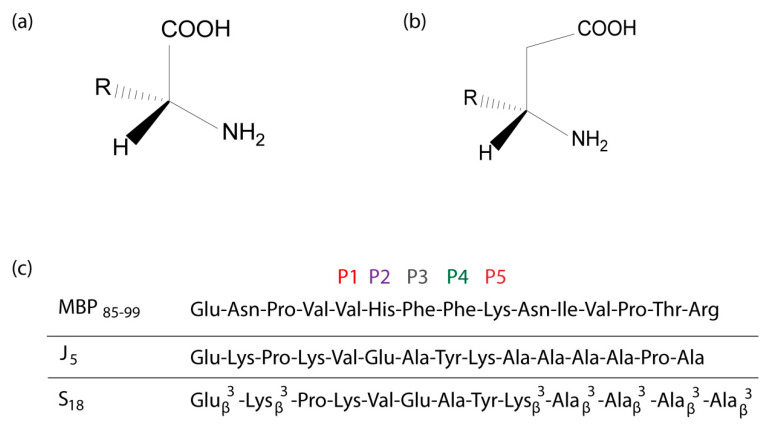
(**a**) α-amino acid, (**b**) Homo-β-amino acid, (**c**) MBP_85-99_, J5 and S_18_ analogue.

The superimposition of the two peptides at the binding site of the trimolecular complex shows that Phe^91^ and Tyr^91^ occupy almost identical areas. However, they induce different conformations to other vicinal amino acids Asn^92^ and Ile^93^, as the phenolic hydroxyl group lies in a relatively hydrophobic environment. Their apparently small structural difference induces a sequence of distinct interactions that determine their fingerprint of biological action. MBP_(85–99)_ is an immuno-dominant epitope of MBP which binds to the MHC haplotype HLA-DR2 and is associated with the pathogenesis of MS. The synthetic 15-mer peptide J5n (Figure 11), was designed and was found to antagonize MBP_(85–99)_ through the binding of MBP_(85–99)_ to soluble HLA-DR2b [73]. The therapeutic efficacy of J5 is limited, probably due to its low biological half-life or bioavailability. The structural features of J5 in relation to its parent (i.e., MBP_(85–99)_) are shown in Figure 11. Phe at position P4 has been replaced with Tyr, Val at position P1 has been retained and His, Phe, and Lys at P2, P3 and P5 have been replaced with Glu, Ala and Lys, respectively.

In another study J5 was derivatized into analogs possessing superior biological half-lives and antagonistic activities. This is achieved by substitution of some of its residues with homo-β-amino acids. S18 (Figure 11), the most active analog, ameliorated symptoms of EAE at least twice more effectively than glatiramer acetate or J5. S18 showed high resistance to proteolysis, which contributed to a delayed clinical onset of disease and prolonged therapeutic benefits [74].

**Figure 12:**
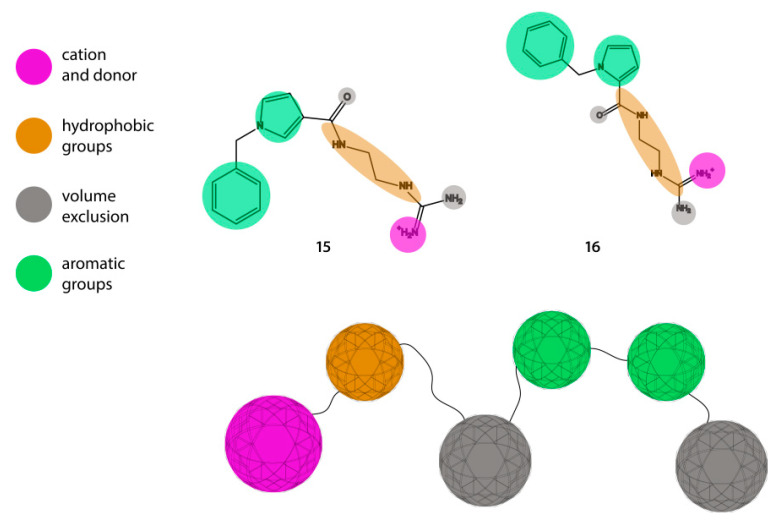
Structure-based pharmacophore derived from ZINC database data.

The conformational analysis studies of MBP_83–96_ epitope led the group of Professor T. Tselios to search for the mining and synthesis of non-peptide mimetic molecules. In particular, they sought molecules that inhibit the trimolecular complex formation and consequently the proliferation of activated T-cells. They generated a structure-based pharmacophore and used ZINC as a chemical database to extract candidates (Figure 12). Semi-empirical and density functional theory (DFT) methods were performed to predict the binding energy between the proposed non-peptide mimetics and the TCR. From the six synthesized molecules the following 15 and 16 were the most promising as they inhibited the stimulation of T-cells by the immunodominant MBP_83–99_ from immunized mice [75].

## 3. Conclusions

An extensive effort has been made the last years to explore the conformational properties of key peptides involved in MS. The conformational analysis of the different epitopes, consisting of in silico MD and pharmacophore studies, along with NMR spectroscopy, has led to the rational design of some bioactive non-peptide mimetics and provided some mechanistic input of the agonistic and antagonistic action of ALPs. However, there is still a long way towards the generation of more potent compounds. Interestingly, in a study it was illustrated that the extent of MHC or TCR competition does not successfully predict the EAE treatment [76]. Other routes to treat MS had also limited success [21,77].

**Figure 13:**
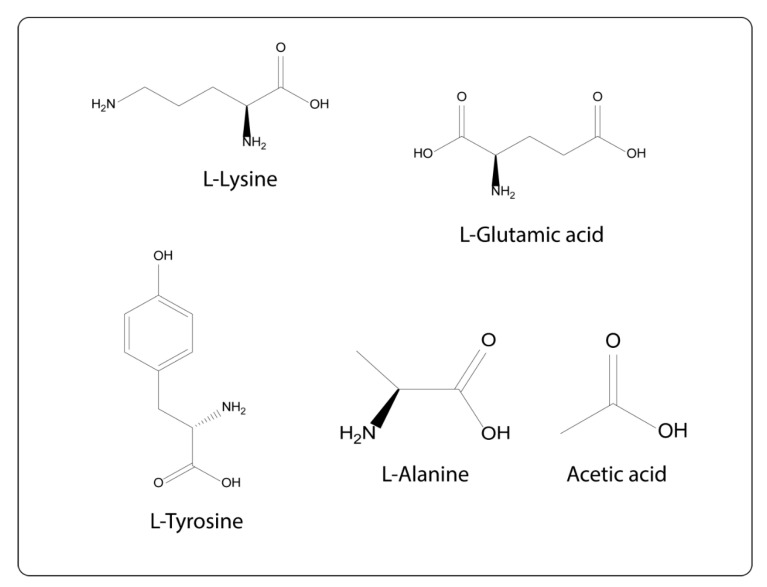
Synthetic peptides of Cop1 structure.

Such an example is the immunomodulatory co-polymer 1 (Copaxone, glatiramer acetate) drug. This contains synthetic peptides composed of nonspecific sequences of four amino acids: L-alanine, L-lysine, L-glutamic acid and L-tyrosine (Figure 13). As its composition is based on the amino acid structure of MBP it exerts an antagonistic action to the 82–100 epitope of MBP [78].

Recently, semi-empirical calculations have been applied to detect peptides associated with MS. It was found that the A_31:01 allele may be associated with the MS disease and the peptide Leu-Ile-Ile-Cys-Tyr-Asn-Trp-Leu-His-Arg may serve as a potential epitope to this allele. This finding must be confirmed by experimental evidence [79].

The multifactorial aspects of MS, especially in its severe state, makes the task of finding a drug against MS tremendously difficult. This must reinforce the efforts in order to advance the progress of understanding and treating the disease.
